# Validation of the patient activation measure in patients at discharge from hospitals and at distance from hospital care in Sweden

**DOI:** 10.1186/s12889-019-8025-1

**Published:** 2019-12-19

**Authors:** Amanda Hellström, Mesfin Kassaye Tessma, Maria Flink, Anna Dahlgren, Kristina Schildmeijer, Mirjam Ekstedt

**Affiliations:** 1 Department of Health and Caring Sciences, Linnaeus University, Kalmar, Sweden; 2 Department of Learning, Informatics, Management, and Ethics, Karolinska Institutet, Stockholm, Sweden; 3 Department of Social Work, Karolinska University Hospital, Stockholm, Sweden; 4 Department of Clinical Neuroscience, Karolinska Institutet, Stockholm, Sweden

**Keywords:** Patient activation measure, Psychometric properties, Rasch measurement model, Validation

## Abstract

**Background:**

The Patient Activation Measure (PAM) is a recognized measure of how active patients are in their care, and has been translated into several languages and cultural contexts. Patient activity, self-care, and health literacy have become increasingly important aspects of health care, and thus reliable measures of these are needed. However, a Swedish translation of PAM is currently lacking. The aim of the study was to translate and assess the validity and reliability of the Swedish PAM-13.

**Methods:**

A self-report questionnaire was handed out to 521 patients at ten medical, geriatric, and surgical wards, and one Virtual Health Room. The Rasch model was employed, using the partial credit model, to assess the functioning of the PAM scale, item fit, targeting, unidimensionality, local independence, differential item functioning (DIF), and person-separation index. Evidence of substantive, content, structural, and external validity was examined.

**Results:**

Of the 521 patients who were consecutively handed a questionnaire, 248 consented to participate, yielding a response rate of 47.6%. The average measure for each category advanced monotonically. The difficulty of the PAM items ranged from − 1.55 to 1.26. The infit and outfit values for the individual items were acceptable. Items 1, 2, and 4 showed disordered thresholds. The mean person location was 1.48 (SD = 1.66). The person-item map revealed that there were no item representations at the top of the scale. The evidence for unidimensionality was ambiguous and response dependency was seen in some items. DIF was found for age. The person separation index was 0.85.

**Conclusion:**

The Swedish PAM-13 was reliable, but was not conclusively found to represent one underlying construct. It seems that the Swedish PAM-13 lacks strong evidence for substantive, content, and structural validity. Although valid and reliable measures of ability for activation in self-care among patients are highly warranted, we recommend further development of PAM-13 before application in everyday clinical care.

## Background

Patients’ active involvement in their own health is a key feature for successful health care. Living with long-term conditions can be highly demanding, and requires that patients manage their symptoms, disabilities, and complex medical regimens [1], as well as taking on the administrative task of coordinating care when multiple care providers are involved [2].

The transition of patients across institutional borders imposes a responsibility on the health care system to engage patients in taking an active role in follow-up treatments and care plans. Discharge from hospital care is an important part of the patients’ preparation to manage their health at home. However, when information is given hastily, patients leave the hospital with an incomplete understanding of their diagnoses, medication changes, and plans of care [3], meaning that they are unprepared for discharge and self-management activities at home [2]. This is especially true for patients living in rural areas where access to specialist care is limited and visits to a health care center are associated with long journeys and significant costs [4]. Demographic changes, with a larger aging population, in addition to the challenges engendered by people living in rural areas, lead to high demands on active self-management skills [5, 6]. In 2004, Hibbard and colleagues introduced a measure of patient activation, i.e., “the knowledge, skill, and confidence to manage one’s health and health care” [7, 8]. A focus on activation recognizes that patients manage their health on their own most of the time, making decisions daily that affect both their health and societal costs [7].

### Patient activation measure (PAM)

The PAM was developed through a series of studies, in order to measure levels of patient activation. The first version, developed in 2004, comprised 22 items [8], and in 2005, a shorter version of 13 items was developed to enhance the feasibility of measuring activation [9]. PAM-13 is a self-report questionnaire and has been validated in various patient groups, such as mental health patients [10], primary care patients [11], patients with neurological problems [12], and patients with chronic diseases [13, 14]. It has been translated into several languages, including Danish [15], Norwegian [10], German [13], Dutch [14], Hebrew [16], and Korean [17].

Each item of the PAM has five response categories with scores from 1 to 4: strongly disagree (1), disagree (2), agree (3), agree strongly (4), and not applicable (no score). The overall score is calibrated into a metric from 0 to 100, where a higher score indicates greater patient activation [18]. The score can be used to assess the level of engagement a patient has. Items #1 and #2 belong to level one, items #3 to #8 to level two, items #9 to #11 level three, and items #12 and #13 to level four. The four levels of patient activation are reached progressively before a patient becomes fully engaged in managing their own health and could be described as an approach to optimizing the patient’s active performance [9]. The levels are: (1) disengaged and overwhelmed; (2) becoming aware but still struggling; (3) taking action; and (4) maintaining behaviours and pushing further. Persons with a high score have a high activation level. The cut-off values that form the four levels of activation on the scale 0–100 have been empirically developed for the English language PAM-13 [18].

Previous studies indicate that patient activation is associated with improved health outcomes and care experiences, reduced health care costs and health care utilization [7, 19, 20]. The first vulnerable period after hospital discharge, or living at distance from health care service, imposes high demands on the patients knowledge, skills and confidence in managing their self-care at home. PAM may be used as a screening tool that reflect the extent to which patients are prepared to implement self-care. However, a validated Swedish PAM is currently lacking. In order to improve the quality of transitional care, and care at home, there is a need for a tool that could be confidently used to identify individual needs for information and self-management support, as well as to evaluate patients’ activation in self-care.

### Translation and adaptation

The translation and adaptation followed the recommendations of the World Health Organization [21]. First, the translation of PAM into a Swedish version was approved by the instrument developer. Two native Swedish speakers (MF and ME) each performed a forward translation from English to Swedish. The two versions were compared and consolidated into one. An expert panel comprising three researchers and three health care workers reviewed the consolidated version and identified wording in need of refinement. The suggestions of the expert panel were reviewed and managed by MF, AD, and ME. A professional translator without knowledge of the original questionnaire received the revised Swedish translation for back-translation. The back-translation was reviewed and compared with the original version by MF, ME, and the instrument developer. The developer raised two concerns, which were addressed. A second expert panel of four persons with experience in health care (two of whom were also experienced in patient participation/activation, one in questionnaire development and one in health care management) provided alternative translation suggestions to increase relevance to Swedish health care. The panel’s suggestions for improvement were harmonized into a new version of the questionnaire by MF and ME. A sample of three patients living with chronic conditions was recruited from patient organizations for cognitive debriefing. MF and ME compiled the final version of the questionnaire based on the input from the patients.

## Method

The aim of the present study was to translate the Patient Activation Measure (PAM-13) into a Swedish version and assess its validity and reliability among patients at discharge from hospital and in primary care, using Rasch analysis.

### Participants and setting

This cross-sectional study was approved by the Regional Ethical Board in Stockholm, Sweden, no. 2014/1498–31/2. Participants were recruited at one Virtual Health Room (VHR), and ten wards (medical, geriatric, and surgical) in five hospitals. One of these was a university hospital, and four were county hospitals representing three different regions of Sweden. The VHR included patients in rural areas of Sweden. The inclusion criteria were patients ≥18 years, who understood Swedish and managed their own health in their private homes. Patients with known psychiatric diagnosis and cognitive impairment were excluded. In total, 521 questionnaires were consecutively handed out by nurses and team leaders at the study sites to patients, at the time of hospital discharge or when they visited the VHR. In all, 248 individuals consented to participate in the study and completed the questionnaires, generating a response rate of 47.6%.

### The questionnaire

Information about the study was given to adult patients, fulfilling the inclusion criteria by the time of hospital discharge, or after the health care service at the VHR. Persons who were willing to participate got a closed envelope comprising the PAM-13-S and questions on socio-demographics (i.e., sex, age, and education), diagnosis, and self-rated health. Education was divided into four ordinal categories, university level being the highest. Diagnosis was divided into six nominal options, and self-rated health was measured on a 5-point scale from excellent to poor. The patients also received a stamped, addressed return envelope. No reminder was sent out. Consent to participation was confirmed by a written consent form that was completed together with the questionnaire (Additional file 1).

### Data analysis

Descriptive statistics were employed to summarize patient characteristics and PAM-13-S.

Rasch analysis was used to evaluate the psychometric properties of PAM-13-S. Data were tested for their fit to the Rasch model using the estimation method Joint Maximum Likelihood Estimation (JMLE) in WINSTEPS, version 4.0 (Linacre, 2017). SPSS for Windows version 25.0 software (SPSS Inc., Illinois, USA) was used to analyse descriptive statistics.

We evaluated the functioning of the PAM-13-S and the presence of theoretically congruent item hierarchies to provide evidence of substantive validity [22–24]. Substantive validity is the extent to which theory explains differences in responses to items [24]. The intended operation of categories and the intended increasing levels of the thresholds (i.e., the points between two adjacent response categories where the conditional probability is equal) across response categories were examined. Item threshold values and category probability curves were evaluated to identify disordered thresholds. If the response categories do not advance monotonically or if disordered thresholds occur, collapsing response categories is suggested to minimize this problem [22, 23]. “Coherence” values examine the empirical relationship between the ratings and the measures. The computation of coherence is given as M - > C (measure implies category %) and C - > M (category implies measure). M - > C reports what percentage of the ratings expected to be observed in a category that are actually observed to be in that category. Forty percent is suggested as an empirically useful level of coherence [22]. We further examined item and person goodness of fit and person-item map to evaluate content validity [25]. To evaluate how data fitted the model, infit mean square error (MnSq) and outfit MnSq were used. For response categories, items, and persons, a fit value (infit and outfit) of 1.0 implies perfect fit to the Rasch model. Fit values lower than 1.0 indicate less variation than expected in responses (response sets in extreme cases). Fit values greater than 1.0 indicate greater variability than expected in responses [26]. Item, person, and response category fit values between 0.5 and 1.5 are acceptable and indicate good fit to the model [9]. The separation between item locations should be > 0.15 logits [27]. Inter-item separation between adjacent items of less than 0.15 logits may indicate redundancy.

Structural validity was addressed by evaluating unidimensionality and local independence [24, 25]. To address unidimensionality, a *principal component analysis of the residuals* (PCAR) was performed [28]. The evaluation of unidimensionality was based on three criteria; at least 50% of the total variance should be explained by the first latent dimension (Rasch dimension) and the first contrast should not have an eigenvalue > 2.0. Disattenuated correlation values close to 1 indicate empirically that the clusters of items are measuring the same thing and that the analysed measure is likely unidimensional [28–30].

Local independence of PAM-13-S items was examined using standardized residual item correlations. Negative or zero standardized residual item correlations suggest that the items reflect local independence [28, 29]. When the number of items is fewer than twenty, the magnitude of the residual item correlation is compared with the average residual correlation for all items [31]. If the correlation between two PAM items is larger than the average correlation of PAM, it indicates that the items on the PAM exhibit local dependency.

In addition, differential item functioning (DIF), (i.e., if the item characteristic curves differ between groups), and person-separation index was investigated. DIF was assessed across the dichotomous categories of age, sex, self-reported general health status, educational level, and main diagnosis. The magnitude of uniform DIF was evaluated using the non-parametric Mantel-Haenszel statistic and a Bonferroni corrected *p* value was used. Linacre recommended that the DIF contrast should be at least 0.5 logits and statistically significant for DIF to be noticeable (*p* ≤ .05).

The person-separation reliability represents the ability of the measure to separate patients into distinct levels of activation. A person reliability of 0.80 with a person separation index of 2.0 is considered acceptable for a scale to distinguish between three or more levels [28]. There is also another analogous estimate of reliability, the Cronbach’s alpha, which should exceed 0.7. Both reliability estimates are included in the results.

## Results

### Participants

The median age of respondents was 70 years, range 20–96 years, and 127 (51.2%) were males. About half of the respondents had primary school education or lower, and one-fifth (19.4%) had a university education. The most common diagnoses were heart failure (21%) and chronic obstructive pulmonary disease (16.9%). The largest group was that of surgical and acute patients with various diagnoses (Table 1). One-hundred-and-twenty-four respondents (50.4%) rated their health as very good or excellent and twelve (4.8%) rated their health as poor (Table 1).

**Table 1:** Demographic and baseline characteristics of the Swedish sample (*n* = 248)

Variables	n (%)	Missing n (%)	PAM total score^b^ Mean (SD)
Sex		1 (0.4)	
*Male*	127 (51.2)		57.3 (13.0)
*Female*	120 (48.4)		60.2 (15.9)
Age^a^ (years)	70	4 (1.6)	
Education		5 (2.0)	
*Less than 9 years*	31 (12.5)		52.5 (16.4)
*Primary school (= 9 years)*	84 (33.9)		56.5 (15.1)
*Secondary school/Vocational training*	80 (32.3)		61.3 (13.9)
*University*	48 (19.4)		62.2 (12.3)
Main diagnosis		10 (4.0)	
*Chronic obstructive pulmonary disease*	42 (16.9)		54.7 (15.8)
*Heart failure*	52 (21.0)		54.3 (13.4)
*Arterial fibrillation/AF*	10 (4.0)		67.4 (14.5)
*Cancer*	36 (14.5)		56.1 (10.7)
*Diabetes mellitus*	6 (2.4)		61.4 (16.8)
*Surgical/Acute*	92 (37.1)		62.3 (14.3)
General health		1 (0.4)	
*Excellent*	42 (17.3)		60.4 (18.2)
*Very good*	82 (33.1)		62.6 (15.6)
*Good*	72 (29.0)		62.7 (13.2)
*Fair*	38 (15.3)		57.1 (13.4)
*Poor*	12 (4.8)		51.7 (14.3)

^a^Median age, ^b^Total score is the calibrated 0–100 metric of the 242 people who completed 10 or more items of PAM

Ranges of missing item responses were between 0.8 and 7.4%. The frequency distribution of the PAM-13-S items is presented in Table 2. All response alternatives were endorsed in all items, but they showed large variation (Table 2). Six people answered ‘strongly agree’ to all items and were excluded from the Rasch analysis since they lacked fit statistics.

**Table 2:** Distribution of response alternatives for the items in the Patient Activation Measure (PAM)

Item	Total n (%)	Strongly disagree n (%)	Disagree n (%)	Agree n (%)	Strongly agree n (%)	Not applicable n (%)	Missing values n (%)
PAM 1	245 (98.8)	2 (0.8)	3 (1.2)	110 (44.4)	130 (52.4)	–	3 (1.2)
PAM 2	246 (98.8)	4 (1.6)	10 (4.0)	133 (53.6)	98 (39.5)	2 (0.8)	1 (0.4)
PAM 3	246 (98.8)	8 (3.2)	33 (13.3)	134 (54.0)	70 (28.2)	–	3 (1.2)
PAM 4	243 (93.5)	8 (3.2)	12 (4.8)	95 (38.3)	117 (47.2)	1 (0.4)	15 (6.0)
PAM 5	246 (99.2)	8 (3.2)	54 (21.8)	113 (45.6)	71 (28.6)	–	2 (0.8)
PAM 6	245 (98.4)	5 (2.0)	21 (8.5)	130 (52.4)	88 (35.5)	2 (0.8)	2 (0.8)
PAM 7	242 (97.2)	3 (1.2)	27 (10.9)	119 (48.0)	92 (37.1)	1 (0.4)	6 (2.4)
PAM 8	245 (98.8)	9 (3.6)	33 (13.3)	128 (51.6)	75 (30.2)	–	3 (1.2)
PAM 9	242 (96.4)	14 (5.6)	63 (25.4)	110 (44.4)	52 (21.0)	–	9 (3.6)
PAM 10	240 (94.8)	17 (6.9)	71 (28.6)	107 (43.1)	40 (16.1)	–	13 (5.2)
PAM 11	236 (92.7)	14 (5.6)	54 (21.8)	118 (47.6)	44 (17.7)	4 (1.6)	14 (5.6)
PAM 12	240 (94.4)	19 (7.7)	77 (31.0)	115 (46.4)	23 (9.3)	7 (2.8)	7 (2.8)
PAM 13	241 (96.1)	19 (7.7)	71 (28.6)	111 (44.8)	37 (14.9)	–	10 (4.0)

### PAM rating

Each response category contained more than ten observations and the average measure for each response category advanced monotonically except for item 1, 2 and 4. Of the four response alternatives (N/A omitted), the response alternative ‘strongly disagree’ was scarcely used by the participants (Table 2). The computation of coherence exceeded 40% for all categories except ‘strongly disagree’, where C - > M was 27%. Inference of ratings-to-measures is generally less successful, as it is below the accepted value 40%, suggesting that the local inference for the PAM-13-S data would improve if response categories 1 and 2 were combined. Therefore, the response categories were reduced to three by post-hoc collapsing of the categories ‘strongly disagree’ and ‘disagree’. The response categories performed well in the PAM-13-S when ‘disagree’ and ‘strongly disagree’ were collapsed. The PSI was acceptable (0.85) and response category infit and outfit values were less than 2 for all response categories.

**Fig. 1:**
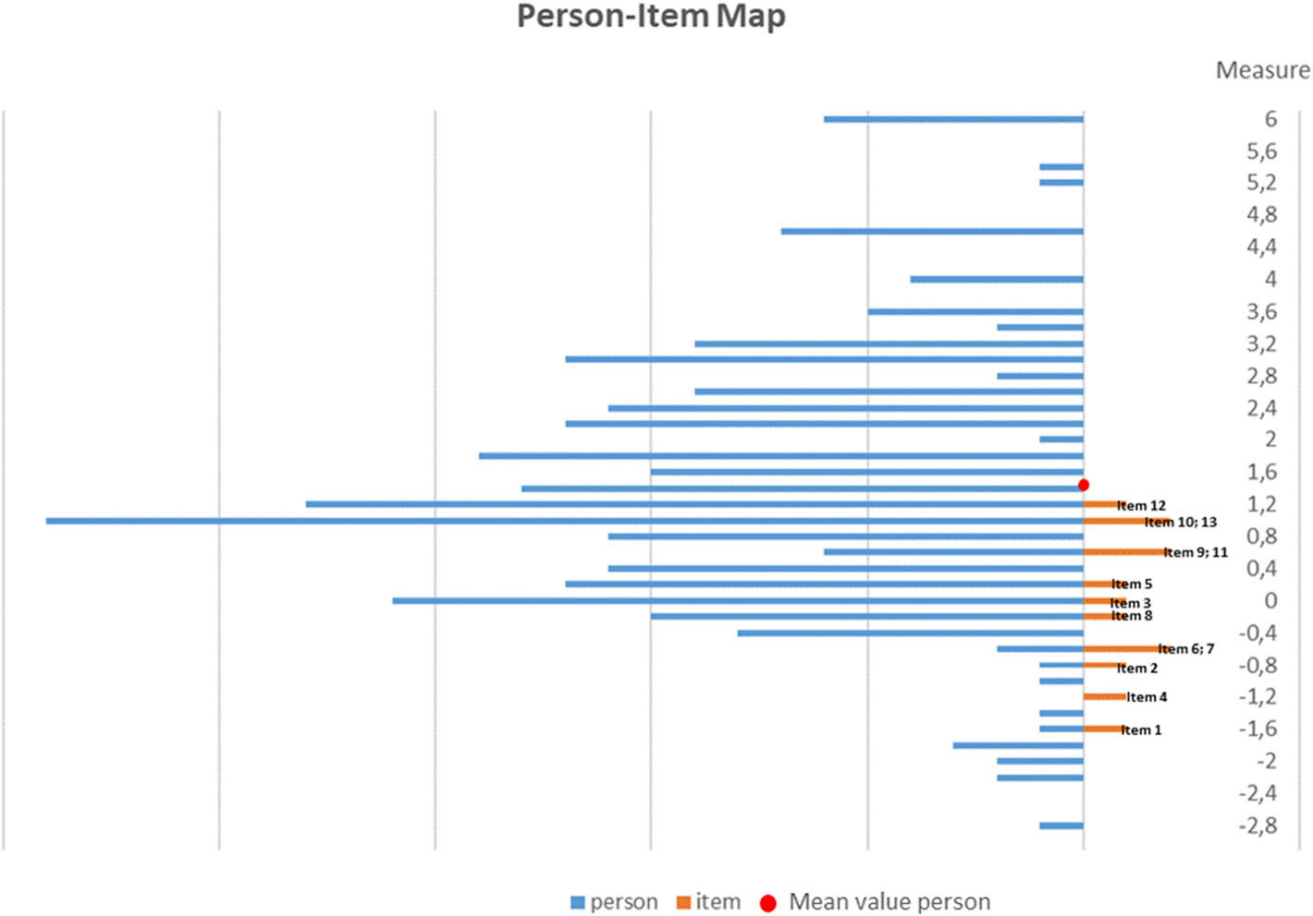
Person-item map of S-PAM-13. The mean location for the item was set at 0. The mean location for person was 1.48 (SD 1.66)

The measures of item difficulty are presented with 95% confidence intervals in Table 3. The item location parameter ranged from a low for item 1, which was the easiest item (logits − 1.55), to a high for item 12, which was the most difficult (logits 1.26). The MnSq values for the 13 PAM items was between 0.81 and 1.28 and thus all items met the criterion 0.5 to 1.5 set for item goodness-of-fit (Table 4). There were 44 (17.7%) persons with outfit MnSq values outside the acceptable range of 0.5 to 1.5 logits (data not shown). However, separation difficulties were seen between adjacent items (Table 3). Separation between item locations should be > 0.15 logits. The Swedish PAM-13 had lower separation difference between items 9 and 11 (difference of 0.01 logits) and items 10 and 13 (0.06 logits). The person-item map (Fig. 1) also illustrates this. Items are arranged by measure from easiest at the bottom (item 1) to hardest at the top (item 12). At the bottom – the negative end of the figure – there are only a few patients and no items, while at the top – the positive end of the figure – there are a lot of patients and no PAM items. The mean person location in this study was 1.48 (SD = 1.66).

**Table 3:** Item, calibration, measure, standard error of the measure, 95% confidence interval, and thresholds

Item number and name	Calibrated PAM score	Measure	SE	95% CI	Threshold 1	Threshold 2	Threshold 3
1. When all is said and done, I am the person who is responsible for taking care of my health*	36.22	−1.55	.13	−1.80 to − 1.30	−3.67	−2.04	1.06
4. I know what each of my prescribed medications do*	39.52	−1.13	.13	−1.38 to −0.88	−3.25	− 1.62	1.48
2. Taking an active role in my own health care is the most important thing that affects my health*	41.5	−0.87	.12	−1.11 to − 0.63	−2.99	− 1.36	1.74
7. I am confident that I can follow through on medical treatments I may need to do at home	43.82	−0.57	.12	−0.81 to − 0.33	−2.69	−1.06	2.04
6. I am confident that I can tell a doctor concerns I have even when he or she does not ask	43.89	−0.56	.12	−0.8 to − 0.32	−2.68	−1.05	2.05
8. I understand my health problems and what causes them	47.38	−0.11	.11	−0.33 to 0.11	−2.23	−0.6	2.5
3. I am confident I can help prevent or reduce problems associated with my health	47.72	−0.07	.11	−0.29 to 0.15	−2.19	−0.56	2.54
5. I am confident that I can tell whether I need to go to the doctor or whether I can take care of a health problem myself	49.7	0.19	.11	−0.03 to 0.41	−1.93	−0.3	2.8
11. I know how to prevent problems with my health	53.61	0.69	.11	0.47 to 0.91	−1.43	0.2	3.3
9. I know what treatments are available for my health problems	53.64	0.70	.11	0.48 to 0.92	−1.42	0.2	3.31
10. I have been able to maintain (keep up with) lifestyle changes, like eating right or exercising	55.91	0.99	.11	0.77 to 1.21	−1.13	0.5	3.6
13. I am confident that I can maintain lifestyle changes, like eating right and exercising, even during times of stress	56.37	1.05	.11	0.83 to 1.27	−1.07	0.56	3.66
12. I am confident I can figure out solutions when new problems arise with my health	57.98	1.26	.11	1.04 to 1.48	−0.86	0.77	3.87

**Table 4:** Infit MnSq, outfit MnSq, and point measure correlation

PAM item	Infit MnSq	Outfit MnSq	Observed correlation	Expected correlation
1	0.97	1.19	.51	.54
2	1.06	1.08	.53	.59
3	1.00	1.03	.63	.64
4	1.18	1.21	.59	.58
5	1.20	1.25	.60	.65
6	1.12	1.12	.55	.62
7	0.89	0.87	.66	.61
8	1.00	0.96	.66	.64
9	1.25	1.28	.60	.66
10	0.81	0.85	.74	.67
11	0.82	0.85	.73	.67
12	0.82	0.85	.69	.68
13	0.85	0.87	.72	.68

A positive mean value for patients indicates that the sample as a whole was located at a higher item difficulty than the average of the scale. It appears that PAM-13-S represents a quantitative continuum from less to more, with a clustering of items between − 1.6 and + 1.3 logits. The map indicates that there are no representations at the top of the scale (beyond + 1.6 logits) among the PAM items. This means that many of the patients do not have any corresponding PAM item.

Unidimensionality of the PAM-13-S was examined using PCAR analysis. The first component explained 48.9% of the total variance and the eigenvalues of the first contrast was > 2.0. The two items with the strongest positive loadings on the first contrast were item 1 (0.60) and item 2 (.56). The three items with the strongest negative loadings were item 13 (− 0.57), item 10 (− 0.56), and item 12 (− 0.53). It was found that the disattenuated first contrast person-measure correlations on the item clusters were 0.73 (item cluster 1–3), 0.84 (item clusters 1–2) and 0.94 (item clusters 2–3). Overall, the evidence for unidimensionality is ambiguous.

### Differential item functioning

We did not observe statistically significant differences in DIF for sex. The DIF test for education using the polytomous MH test showed statistically significant differences between educational levels for item 1 (DIF contrast = 1.20 logits, *p* = 0.015) and item 4 (DIF contrast = 0.83 logits, *p* = 0.04; people with < 9 years of education were different from the others). Also, self-reported general health status revealed significant differences for items 1(DIF contrast = − 1.24, *p* = 0.014), 4 (DIF contrast = 0.91, *p* = 0.021), 9 (DIF contrast = − 0.89, *p* = 0.02), and 10 (DIF contrast = 0.68, *p* = 0.03), between the group with poor health and the group with good health. DIF was also found in item 10 between having poor health and having very good health (DIF contrast = 0.82, *p* = 0.034). Main diagnosis was categorized into two major diagnostic groups: long-term illnesses and acute surgical procedures. However, the statistical significance was not retained when using the Bonferroni correction (*p* < 0.0038) for any of the variables above. Dividing the sample into two age groups, 44 to 64 years and 65 years and older, a statistically significant difference was observed for item 8 (DIF contrast = 0.79, *p* = 0.0019), where the lower age group had a higher DIF score, indicating that item 8 (understanding of health problem) is more difficult for that group.

The real person reliability was 0.84 with a separation index of 2.28, while model person reliability was 0.87 with a separation index of 2.63. The unknown reliability was somewhere between those two values. However, since the separation index was > 2 and reliability was higher than 0.8, the PAM-13-S appears sensitive enough to distinguish between those of high activation and those of low activation. The Cronbach’s alpha value was 0.81.

## D**iscussion**

The aim of the study was to translate and assess the psychometric properties of the Swedish version of the PAM-13 using the Rasch model, in a sample of patients with medical and surgical health conditions who have had a recent encounter with a health care provider. In the current study, efforts have been made to briefly report the different types of validity and to examine some threats to the validity of the PAM-13-S in light of the findings. In the literature, it is known that the two major threats that may obscure meaning and interpretation are construct under-representation and construct irrelevance [32]. Gaps and mismatch on the person-item map may be indications of construct under-representation, while model misfit statistics and multidimensionality may be due to construct irrelevance [24, 32, 33].

### Construct under-representation

With regard to PAM-13-S, construct under-representation implies that the measure did not include all PAM items relevant to the construct, as indicated by lack of targeting in the person-item map; this limits the score meaning and interpretation [32]. The comparison of the person location scores with those of the items provides an indication of poor targeting in the Swedish sample. A substantial portion of the patients whose person measures were above 1.3 did not have a corresponding item that matched their activation level (i.e., ability). This may indicate that the items of PAM-13-S are not difficult enough and that the actual level of patient activation cannot be estimated accurately because of the absence of corresponding items [29]. The mismatch can also be seen from the high mean value of the person estimates (1.48; SD 1.66). The fact that the person measures did not spread along the continuum of the logit scale corresponding to the PAM items is a source of concern when we consider the content and substantive validity of the Swedish PAM.

The frequency distribution of the PAM-13-S items indicates that the proportion was high in the “agree” and “strongly agree” response categories. This may reflect that patient activation was high among patients included in the sample. This finding was further strengthened by the results of the person-item map [34]. Poor targeting implies lower reliability and affects the possibilities to differentiate between people along the latent trait continuum and imprecise estimates.

The person-item map revealed a gap between the mean location of the person versus the mean location of the measure. The mean value of the item was set at zero. In a well-targeted measure, mean person location within ±0.5 logit is acceptable [35, 36].

The findings of this study support that the response category structure was acceptable and advanced monotonically with outfit MnSq values less than 2. One of the important requirements of the Rasch model of polytomous data is adequacy of response categories, i.e., properly ordered, well-defined, and mutually exclusive categories [22, 34]. In the Rasch model, a good-fit is expected to show an ordered set of response thresholds *for each item*; it is expected that respondents with high levels of an attribute would give a high-scoring response and those with low levels would give low-scoring responses [37]. Presence of a disordered threshold will compromise the substantive validity of PAM-13-S, since the rating scale does not then function consistently across items [25]. In the present study, there was an uneven spread of responses between response categories, which affected item functioning. Three items (1, 2, and 4) showed disordered thresholds. Since the C - > M for category 1 was only 27%, the inference of ratings-to-measures was generally less successful [22], suggesting that the local inference for PAM-13-S data would be better if the categories “strongly disagree” and “disagree” were combined. A post-hoc collapsing of the categories ‘strongly disagree’ and ‘disagree’ solved the issue for the Swedish PAM. However, this is not unique; the same problem has forced others [12, 13, 38] to collapse the very same response categories in their translations. It appears that PAM-13-S functions better with fewer response alternatives. On the other hand, both the Italian [39] and the Danish [15] translations kept all response categories; despite the Italian version showing disordered thresholds in two items.

The Swedish version of the PAM-13 showed less separation difference than 0.15 logits between items 11 and 9, and items 10 and 13 (0.06 logits). Both the infit and outfit MnSq values for the 13 PAM items fulfilled the criterion for item goodness-of-fit (0.5 to 1.5). Findings from different European countries have demonstrated that PAM-13 has acceptable infit and outfit values [13, 15]. However, inter-item separation less than 0.15 logits between two adjacent items, indicating redundancy [27], has previously been found in both the German [13] and the Italian versions [39].

### Construct irrelevance

The second threat is construct irrelevance, which refers to the presence of unrelated and irrelevant dimensions [32]. Our results indicated lack of substantial evidence for unidimensionality and this may indicate the presence of construct-irrelevant items. Our study revealed that more than 50% of the variance is unexplained and “non-Rasch”. According to Messick, construct-irrelevant easiness and construct-irrelevant difficulty are the two forms of construct irrelevant variance. Construct-irrelevant difficulty refers to the inclusion of some items that make the measure difficult and construct-irrelevant easiness refers to the inclusion of some items that make the measure easy. In our study, 7 out of 13 items had “negative measure values,” indicating that the Swedish patients found it relatively easy to agree with PAM items. It seems that PAM-13-S showed more easy items compared with the German, Danish, and Singaporean versions [13, 15, 38]. In summary, the evidence for unidimensionality is ambiguous and it seems that there is multidimensionality suggesting that classification of the four patient activation levels using the PAM-13-S may be compromised.

### PAM and patient activation levels

Concerns related to PAM-13 scaling arose from our results. The original PAM-13 not only provides a classification based on total score, but the score is also connected to specific items. Level 1 encompasses items 1 and 2, level 2 encompasses items 3–8, level 3 encompasses items 9–11, and, lastly, level 4 encompasses items 12 and 13. The distributions of responses in our study indicated for example that item 3 *(‘I am confident that I can help prevent or reduce problems associated with my health’)* required a higher level of activation (i.e., was more difficult to achieve) than items 4, 6, 7, and 8. Item 4 *(‘I know what each of my prescribed medications does’)* was perceived as easier than item 2 *(‘Taking an active role in my own health care is the single most important thing affecting my health’).* This might affect the classification between levels 1 and 2 in PAM-13-S and create uncertainty about a patient’s actual ability to participate and act. Consistent with the original American version, items 9, 10, 11, 12, and 13 were the most difficult of the scale, yet their order differed slightly in the Swedish version (11, 9, 10, 13, 12). Scaling variations seem to be a common problem with PAM-13 in European countries [13–15], albeit with large inconsistencies between studies. These studies have shown that PAM-13 performed differently in the populations, as item difficulties varied from the original ranking. These discrepancies may be due to differences in health beliefs embedded in different cultures and/or the different self-management needs of various client groups [38]. Hibbard et al. indicated that the degree to which the measure is valid and reliable in different languages and among different cultures is unknown and deserves investigation [9]. Different countries may have differing cultures and their approach to patient activation may vary. Behavior and its cultural bases are different in each country (or even within a country, between groups). PAM studies have shown that patient responses to PAM items could be affected by response style, cultural influence, the meaning of the items, and the health care system [10, 13, 15, 38]. In Singapore, where English is the working language, Ngooi et al. [40] highlighted a possible cultural influence on patient responses from the collectivistic culture, which promotes harmony and avoidance of confrontations. This could have influenced Singaporean patients not to choose extreme response options. A recent study showed weak association between score on the PAM and adherence to treatment. Possibly external factors such as the attitudes of the physician towards treatment and care plan affect the behaviour and decision of the patient. A negative attitude combined with lack of awareness and knowledge among patients may lead to low adherence [41].

Patient activation might be influenced not only by knowledge, skills, and confidence related to self-management, but also by the health system itself [19]. Our findings could be due to specifics of the sample or cultural and contextual differences, as well as differences between the Swedish and American understanding of roles and responsibilities within the health care system. The patient “who is responsible for taking care of their own health” (item 1), is also the person best suited to initiate patient activation at all levels. For example, level 1 of the activation (items 1 and 2) is related to the beliefs of the patient, which are dependent on several factors including past experience, information from health care professionals, advice from other patients, and own perceptions. Thus, a comprehensive understanding is needed of the beliefs of patients, the practices of health care professionals, and the health care system, which can all relate positively or negatively to patient activation. Hence, we need to pay adequate attention to the context, the complexity of the health care system, and the quality of continuity of care after discharge or diagnosis. Given the possibility that culture influences participant responses, we need to ensure that items and response options are relevant and understood as intended [40]. Our findings require further study to provide substantial conclusions.

### Differential item functioning

The findings revealed presence of DIF in some items. However, DIF is only evidence of bias in cases where the factor causing it is irrelevant to the underlying construct. Age, which should not be of relevance for the construct, showed uniform DIF for item 8, i.e., the group showed a consistent systematic difference in their response to the PAM item, across the whole range of the attribute being measured. To support unidimensionality, it is generally accepted that no more than one item on a scale, or 5% of the items, should demonstrate DIF [25]. In addition, both magnitude and significance should be considered when detecting DIF. For item 8, the contrast was 0.79, and the *p* value 0.0019. Small sample sizes could affect the magnitude of DIF and therefore be misleading, while large magnitudes should always be investigated further. A possible shortcoming of the DIF analysis is that Mantel-Haenszel was used, since only uniform DIF can be detected with this approach. Further investigations of non-uniform DIF could be carried out by visual inspection of empirical item-scale regressions, but we refrained from doing that at this point.

### Strengths and limitations

The use of modern test theory (e.g., Rasch measurement model) offers a number of advantages by modelling the relationship of individual items to the construct being measured. The method provides a much richer description of the performance of each item and greater detail on a measure’s precision than the classical test theory (CTT) [42]. The possibility to map person and item relations in the common logits could also be considered a strength of the Rasch model, since the map shows the relations in a meaningful, easy-to-grasp, pictorial form.

Another major strength of the study is the systematic translation using input from patients, researchers, and health care professionals, which ensured that the items were worded in an easily understood and comprehensible way. The present study had a response rate of 47.6%. There is no direct correlation between response rate and study validity. A low response rate merely indicates a greater risk of low validity [43]. We chose not to ask the wards to gather personal data on included patients (or those who declined participation), to make the personnel at the wards more inclined to participate in distribution of questionnaires. Therefore, we were not able to send out reminders. In retrospect, this had implications that could lead to biases. A consequence is that no analysis of missing cases could be carried out. This is a short-coming of the study, since we do not know if certain patient groups were more reluctant to participate or if the staff distributing the questionnaires were more restrictive to some patient groups or at some wards. However, the sample size was deemed sufficient for the purpose of the study and the analyses performed. In this study, analysis of invariance based on greatly diverse patient groups was performed. This can be considered to be one of the strengths of this study, since it has to be demonstrated that invariance in the Rasch model actually holds across different patient groups. Previous research has demonstrated that for polytomous items, larger sample sizes (> 250 subjects) may be needed to ensure stable and robust estimates of item parameters [44, 45]. In addition, the number of items and response scale categories may be relevant in determining requisite sample sizes. It is recommended that there is at least 10 observations per response-category. In the current study there were < 10 observations for the ‘strongly disagree’ in item 1, 2, 6, 7 and 8. The category ‘disagree’ in item 1 also contained less than 10 observations. This could be seen as a weakness. However, considering the mean square statistics for infit and outfit of items, these are within the recommended interval (0.5–1.5). Even applying a more narrow interval between 0.7–1.3 for infit and outfit statistics, as recommended by Smith et al. [46], the item difficulty parameters are stable.

As shown in Table 2, very few endorsed the ‘strongly disagree’ category, which was reflected in disordered thresholds. Properly ordered data is a requirement of the Rasch model, and increasing levels of severity across response categories of the items should be mirrored in data [47, 48]. This forced us to collapse response categories.

As we wanted to gather data from multiple hospital settings in various regions, questionnaire distribution was delegated to nurses and team leaders at different wards. This may have caused a selection bias, in that the professionals might unintentionally have hesitated to invite patients with low health literacy, due to limited time for providing information. This could lead to underrepresentation of patients with low patient activation.

## Conclusions

The Swedish version of PAM-13 had high reliability, but was not found to represent one underlying construct conclusively. Furthermore, the order of items differed from that in the original PAM-13, as seen previously in European studies. It is possible that differences in health care systems and culture influence how patients perceive the items, explaining the varying order of items between countries. Problems with targeting were noticed, which might indicate construct under-representation.

Although there is a great interest in measuring patient activation across Europe [10, 13–15, 39], only a few studies have recommended PAM for use in clinical practice [10, 49, 50]. However, as the Swedish translation revealed problems with response categories and disordered thresholds, further investigation is warranted prior to its use in everyday clinical practice. Future studies on PAM-13 in Sweden need to examine DIF, response dependency, and unidimensionality, and improve targeting. It seems important to include items that reflect higher levels of patient activation, to decrease the item-person mismatch. The short version PAM, with only 13 items, may not be applicable in the Swedish context. We recommend evaluating the use of the long version, PAM-22.

## Supplementary information

**Additional file 1.** The questionnaire used at data collection.

## Data Availability

Please contact the corresponding author for access to data.

## Abbreviations

DIF: Differential Item Functioning
PAM: Patient Activiation Measure
VHR: Virtual Health Room
