# Supplementary material for: Validation of the patient activation measure in patients at discharge from hospitals and at distance from hospital care in Sweden
Source: BMC Public Health. 2019 Dec 19;19:1701. doi: 10.1186/s12889-019-8025-1 (PMC6921492; doi:10.1186/s12889-019-8025-1)
Supplement: Supplementary file 1 — Additional file 1. The questionnaire used at data collection. [file 12889_2019_8025_MOESM1_ESM.docx]

**Background information**

**Check or fill in the option that works best for you.**

1) Are you?

- - Woman
  - Man
  - Other

2) In what year were you born?

Year of birth:

3) What is the highest degree you have completed?

- - Lower than primary school diploma
  - Primary school diploma or equivalent
  - High school diploma or equivalent
  - University degree

4) What diagnosis do you have?

- - COPD (Chronic Obstructive Pulmonary Disease)
  - Heart failure
  - Other, namely:………………………………

5) In general, would you say your health is:

- - Excellent
  - Very good
  - Good
  - Fair
  - Poor

| 6 |  | Disagree strongly | Disagree | Agree | Agree strongly | N/A |
| --- | --- | --- | --- | --- | --- | --- |
| a) | When all is said and done, I am the person who is responsible for taking care of my health |  |  |  |  |  |
| b) | Taking an active role in my own health care is the most important thing that affects my health |  |  |  |  |  |
| c) | I am confident I can help prevent or reduce problems associated with my health |  |  |  |  |  |
| d) | I know what each of my prescribed medications do |  |  |  |  |  |
| e) | I am confident that I can tell whether I need to go to the doctor or whether I can take care of a health problem myself |  |  |  |  |  |
| f) | I am confident that I can tell a doctor concerns I have even when he or she does not ask |  |  |  |  |  |
| g) | I am confident that I can follow through on medical treatments I may need to do at home |  |  |  |  |  |
| h) | I understand my health problems and what causes them |  |  |  |  |  |
| i) | I know what treatments are available for my health problems |  |  |  |  |  |
| j) | I have been able to maintain (keep up with) lifestyle changes, like eating right or exercising |  |  |  |  |  |
| k) | I know how to prevent problems with my health |  |  |  |  |  |
| l) | I am confident I can figure out solutions when new problems arise with my health |  |  |  |  |  |
| m) | I am confident that I can maintain lifestyle changes, like eating right and exercising, even during times of stress |  |  |  |  |  |

**The following questions is about your ability to participate in and take responsibility for your care. Check the option that works for you.**
